# The State of the Art of Robotic Pancreatectomy

**DOI:** 10.1155/2014/920492

**Published:** 2014-05-28

**Authors:** Marco Del Chiaro, Ralf Segersvärd

**Affiliations:** Division of Surgery, Department of Clinical Science, Intervention and Technology (CLINTEC), Karolinska Institutet, Center for Digestive Diseases, Karolinska University Hospital, Huddinge, K53, 14186 Stockholm, Sweden

## Abstract

During the last decades an increasing number of minimally invasive pancreatic resections have been reported in the literature. With the development of robotic surgery a new enthusiasm has not only increased the number of centers approaching minimally invasive pancreatic surgery in general but also enabled the use of this technique for major pancreatic procedures, in particular in minimally invasive pancreatoduodenectomy. The aim of this review was to define the state of the art of pancreatic robotic surgery. No prospective randomized trials have been performed comparing robotic, laparoscopic, and open pancreatic procedures. From the literature one may conclude that robotic pancreatectomies seem to be as feasible and safe as open procedures. The general idea that the overall perioperative costs of robotic surgery would be higher than traditional procedures is not supported. With the current lack of evidence of any oncologic advantages, the cosmetic benefits offered by robotic surgery are not enough to justify extensive use in cancer patients. In contrast, the safety of these procedure can justify the use of the robotic technique in patient with benign/low grade malignant tumors of the pancreas.

## 1. Introduction 

At the Karolinska Institutet, Hans-Christian Jacobeaus made the first laparoscopic procedure in humans in 1910. The Swedish surgeon used a cystoscope for a diagnostic laparoscopy in 17 patients with ascites [1]. The use of diagnostic laparoscopy for the staging of pancreatic cancer was introduced some months later by Bernheim at Johns Hopkins University [2]. However, only in the late 80s diagnostic laparoscopy for staging of pancreatic cancer was used on a more regular basis [3, 4]. In 1992, Shimi et al. published a series of patients undergoing laparoscopic cholecystojejunostomy for treatment of jaundice [5]. At the beginning of the 90s, the development of higher quality laparoscopic instruments, imaging monitors, and improved surgical techniques enabled not only palliative procedures for unresectable pancreatic cancer to be performed but also the first series of pancreatic resections. In 1994, Gagner and Pomp described the first laparoscopic pancreatoduodenectomy [6]. Even if large series of laparoscopic pancreatoduodenectomies, including complex operation associated with vascular resection, have been published today with comparable results to open procedures [7, 8], the safety of laparoscopic pancreatic procedure is mostly limited to distal pancreatic resections and enucleations [9]. The major problems for the spreading of laparoscopic pancreatoduodenectomy are the dissection of retroperitoneal margin, the complicated reconstruction phase (further complicated by laparoscopic instruments), the length of operating time, and the lack of scientifically proved advantages compared to the conventional open technique. With the development of robotic surgery, however, a new enthusiasm in minimally invasive pancreatic surgery and in particular in minimally invasive pancreatoduodenectomy has grown. The aim of this review is to offer an up to date summary of the state of the art of robotic pancreatic surgery.

## 2. Methods 

Search of MEDLINE and PubMed databases was performed using the keywords “robotic pancreatectomy” and “robotic pancreatic surgery” from 1990 to 2013. Additional articles were identified using a manual search. Series with less than 5 procedures performed were excluded by the analysis and reported eventually to describe particular aspects or techniques. An analysis of the result of major robotic pancreatic procedure was performed.

## 3. Results 

### 3.1. Pancreatoduodenectomy (PD)

With the advent of the robotic era, some of the limitations of laparoscopy are overcome. The robot offers advantages in terms of 3D vision, dexterity, and ergonomy [10]. Giulianotti and coworkers described the first robotic PD in 2003 [11]. In this experience the authors showed the feasibility of this procedure with robotic approach with an acceptable morbidity rate (37.5%), but with a very high mortality rate (12.5%). Today several larger series have been reported in the scientific literature [12–23]. Even though one series contains more than 100 patients [12], the number of procedures per center is still quite small, half of them with less than 10 patients (Table 1). The perioperative results of robotic PD are similar to the open procedures described in the literature. The morbidity and mortality rates range from 60% to 0 and from 4% to 0, respectively. The operative time seems to be longer compared to the open procedure and the mean postoperative stay comparable (Table 1). However to date, no prospective randomized trial has been performed comparing the open with the laparoscopic or robotic procedure. Currently, only 4 nonrandomized studies compared the outcome of open and robotic PD [15, 17, 18, 20]. The operation time was significantly shorter in the open procedure in three and robotic in one of these studies. Furthermore, the mean length of stay (LOS) was shorter in the robotic compared to the open group in three of the studies. No statistical significant differences were found in morbidity and mortality comparing the two groups of procedures. Only one paper in the literature compares the mean costs of robotic versus open PD procedures showing excess of € 6200 with the robotic approach [16]. Robotic PD has been performed for both benign and malignant diseases. As shown in Table 2, the rate of R1 resections for malignant diseases ranges from 0 to 27%; these data most probably reflect different definitions of pathological margin assessment. However, the median number of lymph nodes retrieved in some series is not adequate according to current guidelines to treat pancreatic malignancies [23] (Table 2).

### 3.2. Distal Pancreatectomy (DP)

Minimally invasive DPs are today extensively performed around the world for the treatment of pancreatic tumors and some authors even consider this technique “standard of care” [9]. Today, in most cases the traditional laparoscopic technique is preferred to the robotic approach. The reason is that distal pancreatectomies are less complicated procedures compared to PD without a technically demanding reconstructive phase. In the last decade, however, the number of reports of robotic DP has increased. The results of these studies confirm the safety and feasibility of robotic DP [12, 13, 24–28]. No mortality has been reported in the published series. The morbidity ranges from 9 to 72% and length of stay from 4 to 7 days (Table 3). There are no prospective randomized studies comparing the open, laparoscopic, and robotic DP. A retrospective study comparing the robotic to the traditional laparoscopic technique showed that the robotic technique was associated with a significant increase of spleen preservation rate, operative time, and costs [25]. Another study, retrospectively comparing the results of open, laparoscopic, and robotic DP, confirmed that robotic DP was associated with an increased operative time and spleen preservation rate but significant reduction in LOS compared to both laparoscopic and open DP. Interestingly, the costs of the robotic DP, even if no statistical significant differences were found, seemed to be associated with certain cost reduction [28]. Only three studies have reported on robotic DP for malignancy but data regarding R1-rate or number of lymph nodes retrieved are scarcely reported [12, 13, 24–28] (Table 4).

### 3.3. Robotic Pancreatectomies Associated with Vascular Resection

Three reports of small series of patients undergoing pancreatectomy associated with vascular resection for cancer have been found in the literature. In the first paper [29], 5 patients were included. The mean operative time was 392 minutes and the mean intraoperative blood loss 200 mL. One patient developed postoperative complications (20%). In two cases in which a portal vein reconstruction was required, the mean time of superior-mesenteric/portal vein clamping was 22 minutes. The 6-month survival rate was 80%. In the second paper [16] three cases are described, but no details on the perioperative results and outcome are reported. In the 3rd paper [12] four Appleby operations were reported without perioperative mortality, but with 100% morbidity. The mean operative time was 204 minutes.

### 3.4. Others Robotic Pancreatic Procedures

Many case reports, or small series, of robotic pancreatectomy, are reported in the last year's literature. Enucleation of pancreatic tumors seems to be a safe and effective procedure. Zureikat et al. [12] reported 10 cases performed with no perioperative mortality and 50% morbidity. Few cases of central pancreatectomy, probably also for the rare indication for this procedure [30], are reported in the literature. In a recent series of 13 patients robotically treated [12], there was no perioperative mortality, but 100% morbidity. In another retrospective study [31] including five patients treated robotically and 10 patients treated with open central pancreatectomy, no differences were found regarding overall complication rate and perioperative mortality, but the intraoperative blood loss was significantly lower in the robotic group. In contrast the operative time was longer in the robotic group compared to the open procedure. No significant difference was found in the length of hospital stay. A few small series of total pancreatectomies are also reported [12, 32]. In the Zureikat experience [12], 5 patients were analyzed with no postoperative mortality and with 100% of complication rate. In the Giulianotti series [32], there was no perioperative mortality and 2 patients of 5 (40%) developed postoperative complications. In this study the mean operative time was 456 minutes and the mean length of hospital stay was 7 days. Robotic total pancreatectomies associated with autoislet transplantations have also been described for the treatment of chronic pancreatitis [33, 34].

### 3.5. Overall Evaluation of Results of Robotic Pancreatic Surgery

From the analysis of the current literature, robotic pancreatectomies seem to be feasible and as safe as open procedures. In a recent meta-analysis, Zhang et al. [35] showed a statistical significant advantage of robotic surgery compared to open procedures in terms of overall complication rate, reoperation rate, positive resection margin rate, mean hospital stay, and mean intraoperative blood loss. No differences were found in incidence of postoperative pancreatic fistula, postoperative mortality, and operation time. More complicated is the comparison between robotic and laparoscopic procedures for the lack of comparative data.

## 4. Discussion

During the last decade there has been an increasing interest in minimally invasive pancreatic surgery. The use of traditional laparoscopy, mostly limited to DPs and enucleations [9], has increasingly been described in large series of PD in the last years [7]. The introduction of the robot increased the interest in many centers for minimally invasive pancreatic surgery, even in performing more complicated operations. The improved 3-dimensional imaging, the enhanced dexterity, the better visualization and increased magnification, and the improved ergonomics associated with robotic surgery [10] are some of the most important reasons for the development of robotic pancreatic surgery. The robot offers also the possibility of performing a minimally invasive operation in a way much more similar to traditional open surgery compared to the laparoscopic approach. This difference facilitates the work of the surgeon and can reduce the intraoperative stress [36]. Even if no prospective randomized trials are available comparing results of robotic, open, and laparoscopic pancreatic surgery, data from literature show that robotic pancreatectomies can be performed as safe as the open procedures in experienced centers. However, in all these analyses there is an important bias: the retrospective nature of the studies. The advantages of robotic surgery, compared to the open one, seem to be the traditional ones of minimally invasive surgery, that is, decreased intraoperative blood loss and shorter hospital stay. How much these advantages impact the perioperative costs is very difficult to analyze because there are contradictory results in different retrospective comparative studies. Anyway, the general idea that robotic surgery is more expensive than traditional one is not supported by the literature. Even more complicated is the comparison of robotic and laparoscopic pancreatic surgery. Considering the experience reported in performing PD with the two methods, we can say that robotic technique is more suitable to approach major pancreatectomies. In contrast in DPs, it is difficult to identify advantages. The only reasonable explanation for the use of robot in these procedures can be the easier performing of spleen preserving distal resection and the shorter learning curve to approach these operations. The learning curve aspect is also a topic that should be more investigated [37], even considering the institutional impact on reducing costs. The real problem coming out from this literature analysis is that no long term results of these procedures are available. No data comparing survival in cancer patients are available. For this reason, without a strong evidence of oncologic advantages of robotic surgery and with similar short term results, the cosmetic advantages offered by this technique seem to be not enough to justify an extensive use of it without reasonable cost/effectiveness for cancer patients. In contrast, the safety of these procedures can justify the use of the robotic technique in young patients with benign/low grade malignant tumors of the pancreas that can mostly benefit from a cosmetic operation.

## Figures and Tables

**Table 1:** Perioperative results of robotic pancreatoduodenectomy.

Author	Year	Number of patients	Morbidity (%)	Mortality (%)	Mean OT (min)	Mean POS (days)
Zureikat et al. [12]	2013	132	63	3.8	527	10
Giulianotti et al. [13]	2010	60	NR	3.3	421	22
Zeh et al. [14]	2012	50	56	NR	568	10
Buchs et al. [15]	2011	44	36	4.5	444	13
Boggi et al. [16]	2013	34	56	2.9	517	23
Chalikonda et al. [17]	2012	30	30	3.3	476	9.8
Lai et al. [18]	2012	20	50	0	491	14
Chan et al. [19]	2011	8	NR	0	NR	NR
Zhou et al. [20]	2011	8	75	0	718	28
Kendrick and Cusati [21]	2010	8	NR	NR	NR	NR
de Vasconcellos Macedo et al. [22]	2011	5	60	0	640	26
Narula et al. [23]	2010	5	0	0	420	9.6

**Table 2:** Pathology of resected specimens after robotic pancreatoduodenectomy.

Author	Year	Number of patients	Malignant diseases (%)	Mean lymph nodes harvested	R1 (%)
Zureikat et al. [12]	2013	132	80	NR	NR
Giulianotti et al. [13]	2010	60	75	18	11
Zeh et al. [14]	2012	50	74	18	11
Boggi et al. [16]	2013	34	63	32	0
Chalikonda et al. [17]	2012	30	47	13	0
Lai et al. [18]	2012	20	75	10	27
Zhou et al. [20]	2011	8	100	NR	0
de Vasconcellos Macedo et al. [22]	2011	5	40	NR	NR
Narula et al. [23]	2010	5	20	16	0

**Table 3:** Perioperative results of robotic distal pancreatectomy.

Author	Year	Number of patients	Morbidity (%)	Mortality (%)	Mean OT (min)	Mean POS (days)
Zureikat et al. [12]	2013	83	72	0	256	6
Giulianotti et al. [13]	2010	46	NR	NR	NR	NR
Suman et al. [24]	2013	40	40	0	203	5
Daouadi et al. [25]	2013	30	66	0	293	6
Hwang et al. [26]	2013	22	9.1	0	398	7
Kang et al. [27]	2011	20	10	0	348	7
Waters et al. [28]	2010	17	18	0	298	4

**Table 4:** Pathology of resected specimens after robotic distal pancreatectomy.

Author	Year	Number of patients	Malignant diseases (%)	Mean lymph nodes harvested	R1 (%)
Zureikat et al. [12]	2013	83	72	NR	NR
Giulianotti et al. [13]	2010	46	NR	NR	NR
Suman et al. [24]	2013	40	32	NR	NR
Daouadi et al. [25]	2013	30	43	19	0
Hwang et al. [26]	2013	22	0	NR	0
Kang et al. [27]	2011	20	0	NR	NR
Waters et al. [28]	2010	17	0	5	0
